# Are there trends towards compression of morbidity for dementia? Evidences from four international population‐based cohorts

**DOI:** 10.1002/alz.092173

**Published:** 2025-01-09

**Authors:** Leslie Grasset, Amber Yaqub, Pierre Joly, M. Arfan Ikram, Catherine Helmer, Sudha Seshadri, Alexa S Beiser, Frank J. Wolters, Carole Dufouil

**Affiliations:** ^1^ Univ. Bordeaux, INSERM, BPH, U1219, Bordeaux France; ^2^ Department of Epidemiology, Erasmus University Medical Center, Rotterdam Netherlands; ^3^ INSERM U1219 Bordeaux Population Health ‐ University of Bordeaux, Bordeaux France; ^4^ Department of Epidemiology, Erasmus MC Rotterdam, Rotterdam Netherlands; ^5^ Erasmus University Medical Center, Rotterdam, Zuid‐Holland Netherlands; ^6^ Univ. Bordeaux, Inserm, BPH, U1219, Bordeaux France; ^7^ Glenn Biggs Institute for Alzheimer’s & Neurodegenerative Diseases, University of Texas Health Sciences Center at San Antonio, San Antonio, TX USA; ^8^ The Framingham Heart Study, Framingham, MA USA; ^9^ Department of Neurology, University of Texas Health Sciences Center, San Antonio, TX USA; ^10^ Boston University School of Public Health, Boston, MA USA; ^11^ Department of Radiology & Nuclear Medicine and Alzheimer Center, Erasmus MC, Rotterdam Netherlands; ^12^ CHU de Bordeaux, Pole de sante publique, Bordeaux France

## Abstract

**Background:**

Dementia is a major contributor to loss of autonomy in the elderly. Every year spent with dementia results in significant medical and societal costs. Understanding secular trends of life expectancy with and without dementia would enable us to better anticipate future needs. To date, only a few studies have attempted to decipher the scenarios that occurred over the last few decades concerning the life expectancy (LE) with dementia, but heterogeneity in methods hampered comparison of results. A concerted effort for harmonized methods is essential to investigate whether a decline in dementia incidence is also associated with a compression of morbidity, i.e. a lower duration of LE with dementia.

**Methods:**

We leveraged data from four population‐based studies: PAQUID, Three‐City (3C), the Rotterdam Study (RS), and the Framingham Heart Study (FHS). These studies have ascertained dementia cases and vital status over up to 30 years. Five birth cohorts of participants aged 55 years and older were included in the analysis: 1910‐1915, 1915‐1920, 1920‐1925, 1925‐1930, and 1930‐1935. Illness‐death models were used to compute risk of dementia, risk of death among participants with and without dementia, total LE, dementia‐free LE and LE with dementia. We used age as the time scale and models were adjusted for sex.

**Results:**

Results displayed relate to French cohorts. We included 11450 participants, who were dementia‐free at baseline, from both PAQUID and 3C studies (mean age 75 years, 60% female). On average, the follow‐up duration was 11 years and 2003 incident dementia cases were identified. Overall risks of dementia and risk of death decreased by respectively 44% and 47% in participants from birth cohort 1930‐1935, compared to the 1910‐1915 one. From birth cohorts 1910‐1915 to 1930‐1935, total LE at age 70 rose from 16.3 to 20.3 years, and dementia‐free LE rose from 14.2 to 18.5 years. The risk of death among participants with dementia remained stable.

**Conclusion:**

Results from French cohorts showed an increase in dementia‐free LE over 20 years, while LE with dementia remained stable, which indicates a compression of morbidity. We are replicating these analyses in RS and FHS, and will combine results.

